# Supplementary material for: Unilateral Mitochondrial–Hemodynamic Coupling and Bilateral Connectivity in the Prefrontal Cortices of Young and Older Healthy Adults
Source: Bioengineering (Basel). 2023 Nov 20;10(11):1336. doi: 10.3390/bioengineering10111336 (PMC10669330; doi:10.3390/bioengineering10111336)
Supplement: Supplementary file 1 [file bioengineering-10-01336-s001.zip › bioengineering-2623119-supplementary.pdf]

Unilateral Mitochondrial-Hemodynamic Coupling and Bilateral Connectivity in the Prefrontal Cortices of Young and Older Healthy Adults

Claire Sissons[1], Fiza Saeed[1], Caroline Carter[1], Kristen Kerr[2], Sadra Shahdadian[1], Kathy Lee[2], and Hanli Liu[1,*]

[1] Department of Bioengineering, University of Texas at Arlington, Arlington, TX, 76019, USA
[2] School of Social Work; University of Texas at Arlington, Arlington, TX, 76019, USA
[*] Correspondence: hanli@uta.edu

Supplementary Materials

SA. Decomposition of a Δ[HbO] Time Series into Three Frequency Bands

Figure S1(a) below shows an example of the Δ[HbO] time series from one of the two-channel bbNIRS datasets of a subject. Three curves in Figure S1(b) were obtained after applying a Butterworth band-pass filter to the trace in Figure S1(a) using separate bandpass filters in endogenic (E: 0.005–0.02 Hz), neurogenic (N: 0.02–0.04 Hz), and myogenic (M: 0.04–0.2 Hz) frequency ranges. This set of figures illustrates how different infraslow oscillation components contribute to the composition of the wideband (0.005–0.2 Hz) original signal, as shown in Figure S1(a).

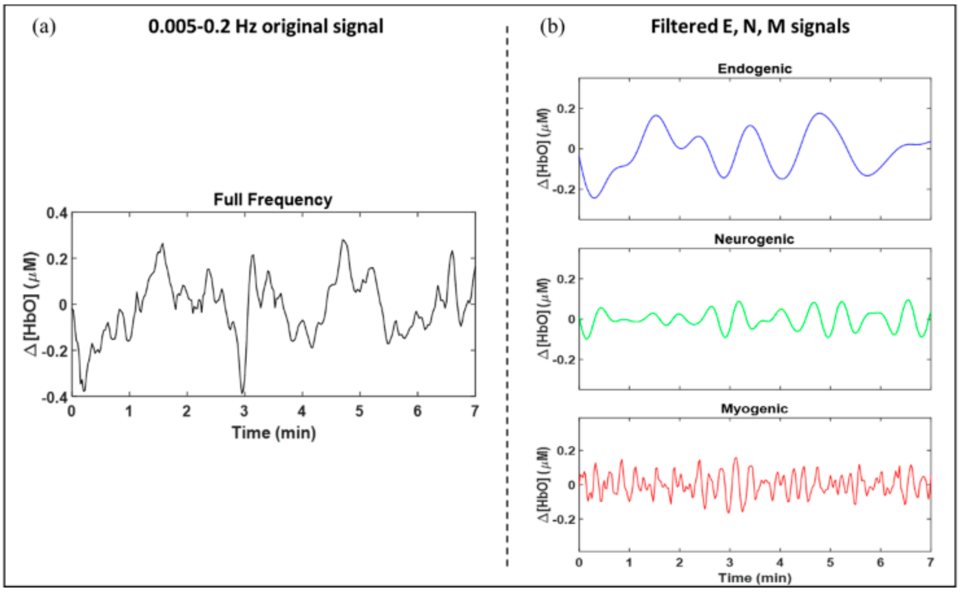

Figure S1 (a) A 7-min time series of Δ[HbO] derived from one of the 2-channel bbNIRS data sets from a random participant. The three panels in (b) on the right were obtained after Butterworth band-pass filtering of the original signal in the three predefined E/N/M bands, namely, 0.005-0.02 Hz, 0.02-0.04 Hz, and 0.04-0.2 Hz, respectively.

SB. Theoretical foundation for conversion from ΔOD(t, λ) to Δ[HbO](t, λ) and Δ[CCO](t, λ)

Methods to quantify changes in concentrations of oxygenated hemoglobin (Δ[HbO]), deoxygenated hemoglobin (Δ[HHb]), and redox-state cytochrome c oxidase (Δ[CCO]) have been developed and reported [10, 33-35]. A brief review is provided below for general readers who wish to understand the theoretical foundation and processing methods in depth.

Figure S2 illustrates graphically the processing steps, which are described below.

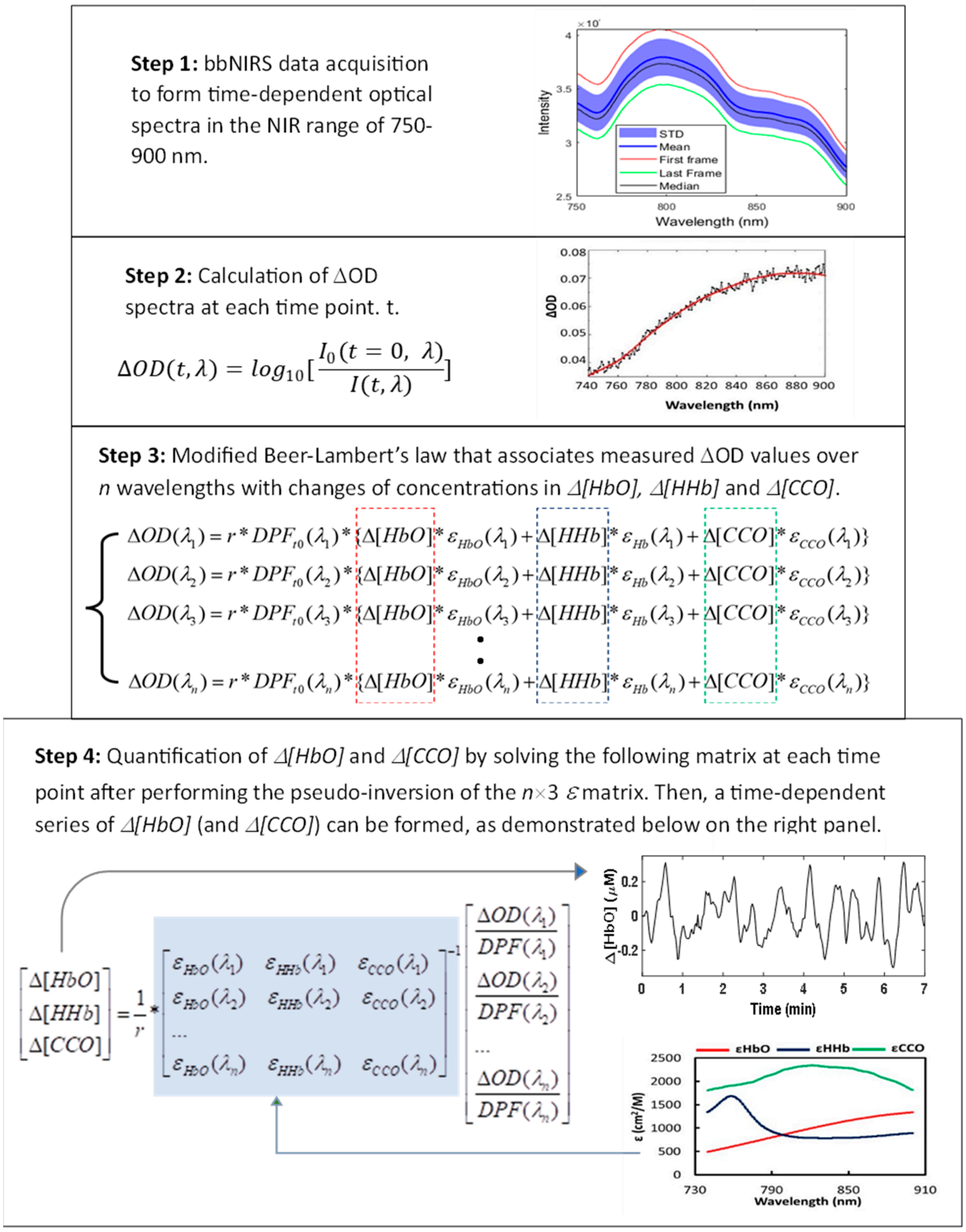

Figure S2. A data processing flow chart used to quantify Δ[HbO], Δ[HHb], and Δ[CCO] from raw bbNIRS data.

Steps 1 and 2:

A broadband near-infrared spectroscopy (bbNIRS) system provides measurements of optical spectra at different times (t), as expressed I(t, λ). A relative optical density spectrum, ΔOD(t, λ), can be defined and calculated at each wavelength λ as [10, 33]:

$$\Delta OD(t,\lambda) = log_{10}[\frac{I_0(t=0,\lambda)}{I(t,\lambda)}], \quad (1)$$

where $I_0(t=0, \lambda)$ can be the baseline spectrum at time *t*=0 or an average of several initial baseline spectral readings (i.e., the first two spectra collected in each experiment), and I(t, λ) represent time-varying spectra acquired at each time point throughout the entire experiment.

Step 3:

The estimations of Δ[HbO] and Δ[CCO] from raw spectral data taken with bbNIRS throughout the experiment were based on modified Beer-Lambert's law [10, 33-35], which offers a quantitative relationship of ΔOD(λ) on Δ[HbO], Δ[HHb], and Δ[CCO] at each wavelength, λ, at each time point, with a wavelength-dependent path-length, L(λ). Furthermore, ΔOD(λ)/L(λ) can be expressed as a sum of optical absorbance contributed by Δ[HbO], Δ[HHb], and Δ[CCO] components, as given below:

$$\begin{bmatrix} \frac{\Delta OD(\lambda_1)}{L(\lambda_1)} \\ \frac{\Delta OD(\lambda_2)}{L(\lambda_2)} \\ \frac{\Delta OD(\lambda_3)}{L(\lambda_3)} \\ \cdots \\ \frac{\Delta OD(\lambda_n)}{L(\lambda_n)} \end{bmatrix} = \Delta[HbO]* \begin{bmatrix} \varepsilon_{HbO}(\lambda_1) \\ \varepsilon_{HbO}(\lambda_2) \\ \varepsilon_{HbO}(\lambda_3) \\ \cdots \\ \varepsilon_{HbO}(\lambda_n) \end{bmatrix} + \Delta[HHb]* \begin{bmatrix} \varepsilon_{HHb}(\lambda_1) \\ \varepsilon_{HHb}(\lambda_2) \\ \varepsilon_{HHb}(\lambda_3) \\ \cdots \\ \varepsilon_{HHb}(\lambda_n) \end{bmatrix} + \Delta[CCO]* \begin{bmatrix} \varepsilon_{CCO}(\lambda_1) \\ \varepsilon_{CCO}(\lambda_2) \\ \varepsilon_{CCO}(\lambda_3) \\ \cdots \\ \varepsilon_{CCO}(\lambda_n) \end{bmatrix}, \quad (2)$$

where Δ[HbO], Δ[HHb] and Δ[CCO] are relative concentration changes of HbO, HHb and CCO respectively; $\varepsilon_{HbO}(\lambda)$, $\varepsilon_{HHb}(\lambda)$ and $\varepsilon_{CCO}(\lambda)$ represent the extinction coefficients at each wavelength of HbO, HHb and CCO, which can be found in ref. [32]; L(λ) is a wavelength dependent variable that denotes the effective pathlength of the detected photons through tissues at each wavelength. Furthermore, according to photon diffusion theory , L(λ) can be expressed as:

$$\begin{bmatrix} L(\lambda_1) \\ L(\lambda_2) \\ L(\lambda_3) \\ \cdots \\ L(\lambda_n) \end{bmatrix} = r* \begin{bmatrix} DPF(\lambda_1) \\ DPF(\lambda_2) \\ DPF(\lambda_3) \\ \cdots \\ DPF(\lambda_n) \end{bmatrix}, \quad (3)$$

where r is a constant that denotes the source-detector distance. In this study, we used source detector separation of 3 cm, so r=3. The wavelength dependence of L(λ) is caused by a wavelength-dependent differential pathlength factor, DPF(λ).

Step 4:

By substituting Eq. (3) into Eq. (2) for multiple wavelengths, the estimation of Δ[HbO], Δ[HHb] and Δ[CCO] can be expressed as follows:

$$\begin{bmatrix} \Delta[HbO] \\ \Delta[HHb] \\ \Delta[CCO] \end{bmatrix} = \frac{1}{r}* \begin{bmatrix} \varepsilon_{HbO}(\lambda_1) & \varepsilon_{HHb}(\lambda_1) & \varepsilon_{CCO}(\lambda_1) \\ \varepsilon_{HbO}(\lambda_2) & \varepsilon_{HHb}(\lambda_2) & \varepsilon_{CCO}(\lambda_2) \\ \cdots & & \\ \varepsilon_{HbO}(\lambda_n) & \varepsilon_{HHb}(\lambda_n) & \varepsilon_{CCO}(\lambda_n) \end{bmatrix}^{-1} \begin{bmatrix} \frac{\Delta OD(\lambda_1)}{DPF(\lambda_1)} \\ \frac{\Delta OD(\lambda_2)}{DPF(\lambda_2)} \\ \cdots \\ \frac{\Delta OD(\lambda_n)}{DPF(\lambda_n)} \end{bmatrix}. \quad (4)$$

In order to accurately solve Δ[HbO], Δ[HHb] and Δ[CCO] using Eq. (4), we would need to know *DPF(λ)* in the wavelength range of our measurements. It is known that appropriate or accurate selection/estimation of wavelength-dependent DPF is crucial for accurate estimation of chromophore concentrations[10, 33]. In this study, DPF(λ) values were assumed to be time-invariant because of given stable brain optical properties. Based on diffusion theory with the semi-infinite boundary geometry [10], DPF(λ) can be determined by

$$DPF(\lambda) = \frac{\sqrt{3\mu_s'(\lambda)}}{2\sqrt{\mu_a(\lambda)}} * \frac{r\sqrt{3\mu_a(\lambda)\mu_s'(\lambda)}}{r\sqrt{3\mu_a(\lambda)\mu_s'(\lambda)}+1} \tag{5}$$

where $\mu_a(\lambda)$ and $\mu_s'(\lambda)$ are the estimated absorption and reduced scattering coefficients across the wavelength range of interest.

Values of $\mu_a(\lambda)$ and $\mu_s'(\lambda)$ were measured using a tissue oximeter (OxiplexTS, ISS) that operates in the frequency-domain. This device provides readings of μ_a and μ_s' values at 750 nm and 830 nm, as well as absolute concentrations of [HbO] and [HHb]. However, to obtain $\mu_s'(\lambda)$ values across the entire range of wavelengths from 780-900 nm, we used Mie theory to interpolate and extrapolate the two measured μ_s' values at 750 nm and 830 nm. Mie theory is typically represented by $k\lambda^{-b}$, where k and b were determined by fitting this equation to both μ_s' values at 750 nm and 830 nm [32]. In addition, absorption coefficients in the same wavelength range (780-900 nm) were estimated based on [HbO] and [HHb] measured by the same tissue oximeter [10].

After combining the measured ΔOD(λ) values across the measurement period and empirical μa(λ) and μs'(λ) values of the human forehead [10, 32], we were able to solve eq. (4) at each measurement time point using MATLAB, achieving temporal series of Δ[HbO], Δ[HHb] and Δ[CCO] under respective experimental conditions, as shown in Step 2 of Fig. 3 in the main paper. Specifically, our calculations covered the spectral range of 780-900 nm with a total of 121 wavelengths.

SC. Steps for Frequency-Domain Data Analysis of Prefrontal Δ[HbO] and Δ[CCO] at Rest

Figure S3 shows a detailed flow chart and demonstrates the frequency analysis steps for a pair of signals, namely, Δ[HbO] and Δ[CCO]. These steps consist of two major functions available in the FieldTrip toolbox [38, 39], namely, "ft_freqanalysis" for amplitude and phase quantifications of each signal, and "ft_connectivityanalysis" for coherence quantification between the two signals.

As illustrated in Figure S3, the input for function "ft_freqanalysis" can be a single time series of two signals, followed by frequency-domain analysis using the multi-taper method (mtm) with *k* tapers [8, 35, 36]. In this step, each tapered time series is subjected to a fast Fourier transform (FFT) to obtain the first set of outputs for this function. A total of k tapers results in k sets of complex numbers with their respective amplitudes and phases in the frequency range of the signal. Next, the mtm-based power spectral density (mtm-PSD) and spectral power are obtained for the input signal by averaging k sets of spectral powers. Accordingly, the respective spectral amplitude (SA) is calculated by taking the square root of the mtm-derived spectral power of the input time series (i.e., Δ[HbO] or Δ[CCO]) in the frequency band of interest. These steps are outlined by blue and orange boxes in Figure S3 for Δ[HbO] and Δ[CCO], respectively.

In addition, the outputs of *k* complex members from "ft_freqanalysis" are used as the inputs for function "ft_connectivityanalysis." This function quantifies the coherence index between the *i*th tapered output of the first signal and the corresponding (i.e., the *i*th) tapered output of the second signal. Each spectral coherence between a pair of temporal signals with the same *k*th taper is averaged over all *k* tapers, leading to the quantification of bilateral connectivity (i.e., $bCON_{HbO}$ and $bCON_{CCO}$) and unilateral coupling (i.e., $uCOP_{HbO-CCO}$) in all three E/N/M frequency bands, as marked by the three green boxes in the figure.

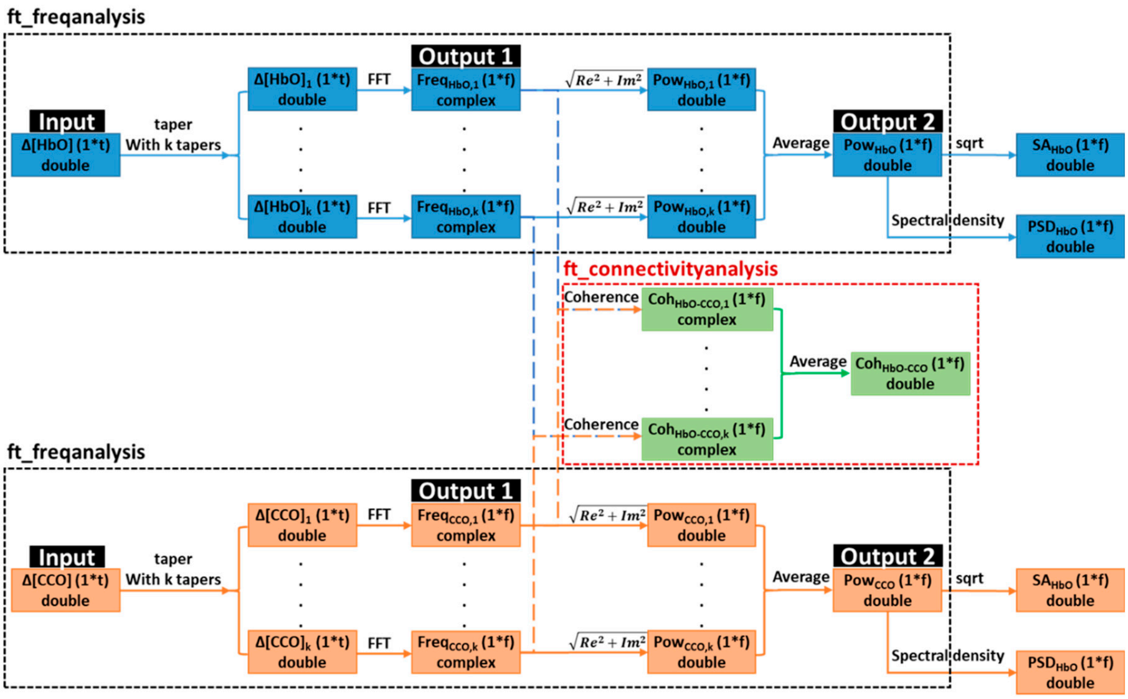

Figure S3 Schematic flow chart of spectral analysis for the quantification of coherence. For demonstration, two time series, Δ[HbO] and Δ[CCO], are used as separate input signals with a time period of 't'. Blue and orange blocks represent frequency analysis steps operated on signal 1 (i.e., Δ[HbO]) and signal 2 (i.e., Δ[CCO]), using "ft_freqanalysis" function (outlined by black dashed boxes). The word of "double" and "complex" indicates a real number with double precision and a complex number, respectively. Furthermore, green blocks represent connectivity analysis steps operated on the frequency-domain outputs of the two signals, using "ft_connectivityanalysis" function (red dashed box).

SD. Comparisons of bCON and uCOP metrics with Eyes-Open and Eyes-Closed Conditions in Older Adults

Table S1. Comparisons of bCON and uCOP metrics with eyes-closed and eyes-open conditions from older adults (n=24)

Frequency Bands	Eyes Closed (n = 24)	Eyes Open (n = 24)	p-value (paired t-test)
**(a) Comparisons of $bCON_{HbO}$ for eyes-open and eyes-closed conditions**			
Endogenic	0.73 ± 0.17	0.72 ± 0.17	0.78
Neurogenic	0.79 ± 0.12	0.74 ± 0.15	0.16
Myogenic	0.58 ± 0.10	0.56 ± 0.13	0.46

**(b) Comparisons of $bCON_{CCO}$ for eyes-open and eyes-closed conditions**			
Endogenic	0.43 ± 0.14	0.44 ± 0.18	0.77
Neurogenic	0.36 ± 0.14	0.36 ± 0.17	0.95
Myogenic	0.35 ± 0.05	0.36 ± 0.06	0.78
**(c) Comparisons of $uCOP_{left}$ for eyes-open and eyes-closed conditions**			
Endogenic	0.47 ± 0.19	0.53 ± 0.20	0.11
Neurogenic	0.41 ± 0.14	0.42 ± 0.18	0.81
Myogenic	0.44 ± 0.06	0.43 ± 0.11	0.86
**(d) Comparisons of $uCOP_{right}$ for eyes-open and eyes-closed conditions**			
Endogenic	0.45 ± 0.14	0.48 ± 0.15	0.24
Neurogenic	0.38 ± 0.15	0.41 ± 0.13	0.32
Myogenic	0.47 ± 0.07	0.45 ± 0.09	0.18

SE. Gender comparisons in bCON and uCOP metrics in young adults

Table S2. Comparisons of gender differences in bCON and uCOP metrics in young adults (n=26)

Frequency Bands	Males (n =14)	Females (n =12)	p-value (two-sample t-test)	Cohen's d
**(a) Gender difference in $bCON_{HbO}$**				
Endogenic	0.78 ± 0.18	0.69 ± 0.25	0.052	N/A
Neurogenic	0.80 ± 0.16	0.74 ± 0.19	0.10	N/A
Myogenic	0.85 ± 0.10	0.80 ± 0.12	0.006*	0.46
**(b) Gender difference in $bCON_{CCO}$**				
Endogenic	0.35 ± 0.25	0.36 ± 0.24	0.50	N/A
Neurogenic	0.31 ± 0.19	0.32 ± 0.23	0.67	N/A
Myogenic	0.15 ± 0.09	0.15 ± 0.07	0.79	N/A
**(c) Gender difference in $uCOP_{left}$**				
Endogenic	0.36 ± 0.26	0.34 ± 0.22	0.76	N/A
Neurogenic	0.33 ± 0.21	0.31 ± 0.21	0.75	N/A
Myogenic	0.26 ± 0.14	0.24 ± 0.11	0.76	N/A
**(d) Gender difference in $uCOP_{right}$**				
Endogenic	0.37 ± 0.21	0.38 ± 0.21	0.50	N/A
Neurogenic	0.23 ± 0.17	0.28 ± 0.17	0.08	N/A
Myogenic	0.24 ± 0.12	0.20 ± 0.09	0.015*	0.44
